# Comparison of the effects of 7.2% hypertonic saline and 20% mannitol on whole blood coagulation and platelet function in dogs with suspected intracranial hypertension - a pilot study

**DOI:** 10.1186/s12917-017-1108-2

**Published:** 2017-06-19

**Authors:** Ivayla D. Yozova, Judith Howard, Diana Henke, Daniel Dirkmann, Katja N. Adamik

**Affiliations:** 1 Institute of Veterinary, Animal and Biomedical Sciences, Massey University, Private Bag 11-222, Palmerston North, 4442 New Zealand; 2 Clinical Diagnostic Laboratory, Department of Clinical Veterinary Medicine, Vetsuisse Faculty, University of Bern, Laenggassstrasse 124, 3012 Bern, Switzerland; 3 Division of Neurological Sciences, Vetsuisse Faculty, University of Bern, Laenggassstrasse 128, 3012 Bern, Switzerland; 4 Clinic for Anesthesiology and Intensive Care, Essen University Hospital, Hufelandstraße 55, 45122 Essen, Germany; 5 Division of Small Animal Emergency and Critical Care, Department of Clinical Veterinary Medicine, Vetsuisse Faculty, University of Bern, Laenggassstrasse 128, 3012 Bern, Switzerland

**Keywords:** Intracranial hypertension, Osmotherapy, Hypertonic saline, Mannitol, Hemostasis, Thromboelastometry, Platelet function

## Abstract

**Background:**

Hyperosmolar therapy with either mannitol or hypertonic saline (HTS) is commonly used in the treatment of intracranial hypertension (ICH). In vitro data indicate that both mannitol and HTS affect coagulation and platelet function in dogs. The aim of this study was to compare the effects of 20% mannitol and 7.2% HTS on whole blood coagulation using rotational thromboelastometry (ROTEM®) and platelet function using a platelet function analyzer (PFA®) in dogs with suspected ICH. Thirty client-owned dogs with suspected ICH needing osmotherapy were randomized to receive either 20% mannitol (5 ml/kg IV over 15 min) or 7.2% HTS (4 ml/kg IV over 5 min). ROTEM® (EXTEM® and FIBTEM® assays) and PFA® analyses (collagen/ADP cartridges) were performed before (T_0_), as well as 5 (T_5_), 60 (T_60_) and 120 (T_120_) minutes after administration of HTS or mannitol. Data at T_5_, T_60_ and T_120_ were analyzed as a percentage of values at T_0_ for comparison between groups, and as absolute values for comparison between time points, respectively.

**Results:**

No significant difference was found between the groups for the percentage change of any parameter at any time point except for FIBTEM® clotting time. Within each group, no significant difference was found between time points for any parameter except for FIBTEM® clotting time in the HTS group, and EXTEM® and FIBTEM® maximum clot firmness in the mannitol group. Median ROTEM® values lay within institutional reference intervals in both groups at all time points, whereas median PFA® values were above the reference intervals at T_5_ (both groups) and T_60_ (HTS group).

**Conclusions:**

Using currently recommended doses, mannitol and HTS do not differ in their effects on whole blood coagulation and platelet function in dogs with suspected ICH. Moreover, no relevant impairment of whole blood coagulation was found following treatment with either solution, whereas a short-lived impairment of platelet function was found after both solutions.

## Background

Osmotherapy is commonly used in the treatment of intracranial hypertension (ICH) due to a variety of causes, including head trauma, intracranial neoplasia, infection or hemorrhage, and status epilepticus [1]. The principle goal of osmotherapy is to shift fluid from the intracellular into the extracellular compartment using intravenous hyperosmolar agents, thereby reducing brain edema and improving cerebral perfusion pressure [2]. Although 10–20% mannitol is considered the gold standard hyperosmolar agent in the treatment of ICH [3], mannitol-induced osmotic diuresis may cause hypovolemia and reduction in cerebral perfusion pressure [1]. In recent years, 3.0–7.5% hypertonic saline (HTS) has gained popularity in the treatment of ICH [4, 5] as it has less pronounced diuretic effects and therefore does not cause hypovolemia [1, 6]. Indeed, in the face of hypovolemic shock and traumatic brain injury, HTS provides the advantage of volume expansion, restoring adequate cerebral perfusion pressures, and reducing brain edema, which makes it superior to mannitol in trauma patients with shock [7, 8].

Both mannitol and HTS have been shown to interfere with whole blood coagulation and platelet function [9]. This is in part due to dilutional coagulopathy. Furthermore, 7.2% HTS may directly disturb both fibrin formation and platelet function [10], and mannitol may interfere with coagulation by reducing clot strength [11]. In addition, hyperosmolarity is supposed to lead to impairment of both whole blood coagulation and platelet function [9, 12]. In consequence, the safety of using these agents in patients with ICH and intracranial hemorrhage remains unclear [4, 11, 13]. Previous in vitro studies in humans have demonstrated anticoagulant effects of both mannitol and HTS [9, 14], although one clinical study failed to demonstrate any negative effect on hemostasis using either solution in patients undergoing elective intracranial surgery [15]. Similarly, in vitro studies in dogs demonstrated that both mannitol and HTS have negative effects on coagulation in a dose-dependent fashion, although in vivo studies in dogs in a clinical setting are lacking [16, 17]. As a result, current guidelines for osmotherapy in dogs with ICH are largely extrapolated from experimental data and human literature [18, 19].

The aim of this study was to compare the effects of clinically recommended doses of 20% mannitol and 7.2% HTS on whole blood coagulation using rotational thromboelastometry (ROTEM®) and on platelet function using a platelet function analyzer (PFA®) in dogs with suspected ICH. Based on a previous canine in vitro study, we expected that mannitol would have a greater impact on coagulation than HTS [17]. A second aim was to compare plasma osmolarity after either osmotherapeutic solution. Knowledge of the extent to which these solutions may impact global hemostasis may influence clinical decision-making in the selection of solutions for individual dogs.

## Methods

The study was designed as a prospective, randomized, non-blinded cohort study using client-owned dogs with suspected ICH. The trial was approved the Animal Experiment Committee of the Swiss Federal Veterinary Office (registration number BE 90/13), and informed owner consent was obtained for all dogs.

### Animals

Dogs with suspected ICH requiring osmotherapy were enrolled between March 2013 and March 2016. A tentative diagnosis of ICH was based on history, neurologic examination (performed by a board-certified neurologist) and/or magnetic resonance or computed tomographic imaging studies (evaluated by a board-certified radiologist or neurologist). Criteria to substantiate suspicion of ICH were a forebrain or multifocal localization with severely reduced consciousness and miotic pupils on neurological exam, Cushing triad (irregular respiration, bradycardia, and systolic hypertension), brain herniation or other shifts of brain parenchyma on MRI or CT [20], an elevated resistive index [21], and deterioration of modified Glasgow coma scale scores [22]. The decision to administer osmotherapy was at the clinicians’ discretion. Exclusion criteria were azotemia (plasma creatinine concentration > 140 μmol/L), body weight < 7 kg, age < 6 months or >12 years, presence of diseases known to affect coagulation (hyperadrenocorticism, protein losing enteropathy or nephropathy, and hepatic insufficiency) and administration of non-steroidal anti-inflammatory drugs, osmotherapeutics (mannitol, HTS), artificial colloids, or blood products within two weeks prior to study enrollment. In addition, dogs were excluded if their initial hematocrit was below 0.25 L/L or if platelet counts were below 100 × 10^9^/L, as this may influence results PFA assays [23]. Clinical data collected included patient characteristics (breed, age, gender, and body weight), the underlying disease causing ICH, and any additional crystalloid fluid therapy administered within the measurement period.

### Treatment groups

Osmotherapeutic solutions used in this study were 20% mannitol (Dr. G. Bichsel AG, Interlaken, Switzerland) (osmolarity, 1100 mOsm/L) and 7.2% HTS (Dr. G. Bichsel AG, Interlaken, Switzerland) (osmolarity, 2464 mOsm/L). Dogs were randomized to receive either solution by selecting a sealed envelope containing a numbered card. Dogs received either 1 g/kg (5 ml/kg) mannitol administered over 15 min or 4 ml/kg HTS administered over 5 min using a syringe pump. Additional crystalloid fluid therapy given during the measurement period was at the clinician’s discretion. Animals were excluded if additional osmotherapy was administered during the study measurement period.

### Measurements

Whole blood coagulation was analyzed using ROTEM® (ROTEM®, TEM Innovations GmbH, Munich, Germany), according to the manufacturer’s instructions using methods previously described for canine samples [24]. Two ROTEM® machines were available to enable parallel measurements of T_0_ and T_5_ samples. Clotting activators used were EXTEM® (re-calcification and tissue factor activation) for assessment of the extrinsic pathway and FIBTEM® (tissue factor activation and platelet inhibition with cytochalasin D) for analysis of the extrinsic pathway and qualitative assessment of fibrinogen status (star-tem®, ex-tem®, and fib-tem® reagents, TEM Innovations GmbH, Munich, Germany). Data collected were clotting time (the time from the start of the measurement until the onset of clotting, CT), clot formation time (the time between the onset of clotting and a clot firmness of 20 mm amplitude, CFT), alpha angle (angle between the baseline and a tangent to the clotting curve through the 2 mm CT, α), and amplitude 10 (clot firmness in mm at the amplitude time point of 10 min after CT, A10), and maximum clot firmness (the maximum amplitude of the curve measured in millimeters, MCF). Platelet function was assessed using a PFA-100® analyzer (PFA-100®; Siemens Healthcare Diagnostics AG, Zurich, Switzerland), according to the manufacturer’s instructions using methods previously described for canine samples [23] and collagen/ADP cartridges (Dade PFA Collagen/ADP Test Cartridge, Siemens Healthcare Diagnostics AG, Zurich, Switzerland). Briefly, the time until occlusion of the aperture by platelet plug formation (closure time, Ct_PFA_) was measured. Institutional reference intervals (RI) for ROTEM® and PFA® used in this study were previously established from jugular blood samples of 37 healthy dogs. Hematocrits and platelet counts were measured using a hematology analyzer (Advia® 120, Siemens Healthcare Diagnostics AG, Zurich, Switzerland) and plasma osmolarity was measured using a freezing-point osmometer (Osmometer Type 1, Loeser Messtechnik, Germany).

### Blood sampling

Blood samples were obtained prior to (T_0_), and five (T_5_), 60 (T_60_), and 120 min (T_120_) after the end of osmotherapy administration. At each time point, blood was obtained by careful puncture of the lateral saphenous vein using a 21G butterfly needle connected to a vacutainer system. To minimize contamination of the whole blood samples with tissue factor, the first and third blood samples were collected from the right saphenous vein and the second and fourth from the left saphenous vein. Blood was collected in the following order: one serum vacutainer tube (discard tube), two 3.2% buffered sodium citrate vacutainer tubes (BD Vacutainer 1.8 ml coagulation tube, buffered trisodium citrate 3.2%, BD, Plymouth, United Kingdom) for PFA® and ROTEM® analyses, one heparin tube (Li-Heparin LH/1.3, 1.3 ml tubes, Sarstedt AG, Sevelen, Switzerland) for osmolarity and one K_2_-EDTA tube (K2-EDTA 2 ml tubes, Sarstedt AG, Sevelen, Switzerland) for hematocrit and platelet counts. Blood samples for ROTEM® and PFA® analyses were maintained at room temperature and analyses were started after 30-60 min. Hematocrits, platelet counts and osmolarity were measured within 4 h of sampling. All blood samples were collected and analyzed by the same investigator (IY).

### Statistical analysis

Descriptive statistics were evaluated and continuous variables were assessed for normality using the D’Agostino-Pearson test and by examining normal plots. Differences between groups (mannitol vs HTS) were evaluated using independent samples t-tests and Mann-Whitney tests for normal and non-normal distributed continuous data, respectively, and chi-squared tests were used for categorical data. For comparison between the groups for ROTEM® and PFA® assays, data at T_5_, T_60_ and T_120_ were evaluated as a percentage of the values measured at T_0_ in each dog.

Repeated measures analyses within each group for osmolarity were performed using a Friedman test and post-hoc pair-wise analysis was applied using Wilcoxon tests with Bonferroni correction. Repeated measures analyses within each group for ROTEM® and PFA® measurements were performed using a Skillings-Mack test, which is a generalization of the Friedman test for incomplete blocks when observations are missing arbitrarily. All data were analyzed using statistical software (MedCalc® version 16.4.3, MedCalc® Software bvba, Ostend, Belgium and Statext version 2.7.17, Statext LLC, Wayne NJ, USA) and significance was set at *P* < 0.05 throughout.

## Results

A total of 30 dogs were included in the study (15 dogs in each group). Dogs had a mean age of 6.2 ± 3.4 years and a mean body weight of 25.9 ± 13.6 kg. There were 13 female dogs (5 sexually intact, 8 spayed) and 17 male dogs (8 sexually intact, 9 castrated). No significant difference was found between groups for age (*P* = 0.99), body weight (*P* = 0.31), or gender (*P* = 0.72). Breeds represented were mixed-breed (*n* = 4), French bulldog (*n* = 4), golden retriever (*n* = 4), Labrador retriever (*n* = 3), border collie (*n* = 2) and 1 each of boxer, cocker spaniel, flat coated retriever, fox terrier, German shepherd, great Dane, Jack Russel terrier, Keeshond, Malinois, mudi, Saint Bernard, Tervuren, and white Swiss shepherd. Of the 30 dogs, 24 underwent magnet resonance imaging and 1 dog underwent computed tomography. Underlying diseases were intracranial neoplasia (*n* = 8, mannitol group; *n* = 8, HTS group), intoxication (*n* = 3, mannitol group; *n* = 1, HTS group), head trauma (*n* = 2, mannitol group; *n* = 1, HTS group), meningoencephalitis (*n* = 2, mannitol group), hydrocephalus internus (*n* = 1, HTS group), presumptive hypertensive encephalopathy (*n* = 1, HTS group), and undetermined in the remaining 3 dogs (HTS group).

In some samples, the anticipated analyses could not be carried out due to technical constraints (sample clotting or measurement error). In addition, blood samples of 7 dogs at T_60_ and a further 3 dogs at T_120_ could not be obtained due to euthanasia prior to the measurement time point. The number of samples used for each analysis is presented in Tables 1 and 2. During the two-hour study period, 19 of 30 dogs received intravenous isotonic crystalloid therapy (median, 5 ml/kg; range, 2–20 ml/kg).

**Table 1:** Osmolarity, hematocrit and platelet counts prior to (T_0_), and at 5 (T_5_), 60 (T_60_) and 120 (T_120_) minutes after administration of mannitol and hypertonic saline (HTS)

Variable	Time point	Osmotherapeutic solution and sample size				*P* value
		Sample size	Mannitol	Sample size	HTS	
Osmolarity (RI: 300-310 mOsm/L)	T_0_	15	312 (308–327)	15	311 (309–320)	0.89
	T_5_	15	329 (321–339)*	15	336 (324–340)*	0.96
	T_60_	11	326 (318–341)*	12	324 (317–336)*	0.62
	T_120_	8	327 (316-334)	11	326 (319–334)*	0.59
Hematocrit (RI: 0.39-0.57 L/L)	T_0_	15	0.44 (0.35–0.49)	15	0.41 (0.37–0.49)	0.97
	T_5_	15	0.37 (0.31–0.44)	15	0.37 (0.34–0.42)	0.68
	T_60_	11	0.37 (0.35–0.41)	12	0.38 (0.37–0.42)	0.42
	T_120_	8	0.42 (0.35–0.44)	11	0.40 (0.36–0.42)	0.70
Platelet count (RI: 150-400 × 10^9^/L)	T_0_	15	221 (167–375)	15	237 (173–312)	0.91
	T_5_	15	202 (157–314)	15	202 (179–287)	0.82
	T_60_	11	207 (169–344)	12	177 (134–284)	0.56
	T_120_	8	214 (188–393)	11	205 (141–269)	0.31

Values are shown as median (interquartile range), *RI* reference intervals; * values differing significantly from T_0_ (*P* < 0.05)

**Table 2:** Rotational thromboelastometry and platelet function analysis closure time prior to (T_0_), and at 5 (T_5_), 60 (T_60_) and 120 (T_120_) minutes after administration of mannitol and hypertonic saline (HTS)

Variable	Time point	Osmotherapeutic solution and sample size			
		Mannitol	Sample size	HTS	Sample size
Ct_PFA_ (RI: 42-90s)	T_0_	76 (72-86)	15	90 (75-102)	13
	T_5_	**96 (79-127)**	15	**96 (77-138)**	13
	T_60_	85 (76-96)	11	**98 (70-113)**	12
	T_120_	79 (75-83)	8	78 (71-118)	11
EXTEM^®^ CT (RI: 20-85 s)	T_0_	42 (37-50)	15	42 (35-54)	15
	T_5_	41 (32-45)	15	38 (35-50)	15
	T_60_	38 (36-49)	11	40 (34-45)	12
	T_120_	43 (29-46)	9	41 (36-49)	11
EXTEM^®^ CFT (RI: 55-374 s)	T_0_	84 (59-129)	14	93 (86-111)	15
	T_5_	114 (74-145)	14	115 (102-124)	14
	T_60_	97 (68-105)	10	115 (99-190)	12
	T_120_	98 (77-125)	8	108 (93-147)	11
EXTEM^®^ A10 (RI: 17-69 mm)	T_0_	58 (46-62)	15	54 (49-61)	15
	T_5_	56 (46-58)	15	51 (39-61)	14
	T_60_	57 (53-67)	11	54 (53-67)	12
	T_120_	60 (52-63)	9	55 (44-56)	11
EXTEM^®^ α-angle (RI: 57-86°)	T_0_	73 (61-81)	14	72 (69-75)	15
	T_5_	76 (65-71)	14	67 (64-71)	14
	T_60_	72 (69-79)	10	69 (64-77)	12
	T_120_	69 (65-81)	8	70 (65-75)	11
EXTEM^®^ MCF (RI: 29-75 mm)	T_0_	68 (52-69)	15	66 (59-69)	15
	T_5_	65 (51-67)*	15	62 (51-67)	14
	T_60_	67 (64-74)	11	63 (50-67)	12
	T_120_	68 (61-71)	9	63 (58-65)	11
FIBTEM^®^ CT (RI: 18-72 s)	T_0_	38 (31-44)	15	42 (36-46)	14
	T_5_	36 (32-44)	15	33 (32-39)*	14
	T_60_	39 (33-50)	11	35 (30-36)*	11
	T_120_	40 (32-44)	9	35 (36-42)*	10
FIBTEM^®^ A10 (RI: 2-17 mm)	T_0_	12 (10-21)	15	11 (6-15)	14
	T_5_	11 (10-17)	15	10 (7-13)	14
	T_60_	14 (10-21)	11	10 (9-15)	11
	T_120_	16 (11-17)	9	11 (8-15)	10
FIBTEM^®^ α-angle (RI: 61-88°)	T_0_	75 (65-79)	13	67 (65-74)	10
	T_5_	75 (67-81)	11	72 (70-75)	10
	T_60_	77 (74-79)	8	67 (62-76)	8
	T_120_	76 (64-78)	8	72 (61-77)	6
FIBTEM^®^ MCF (RI: 3-17 mm)	T_0_	14 (9-20)	15	12 (7-16)	14
	T_5_	13 (9-19)*	15	11 (8-13)	13
	T_60_	14 (9-18)*	11	10 (9-15)	11
	T_120_	15 (13-18)	9	12 (9-15)	10

*Ct*_*PFA*_ platelet closure time, *CT* clotting time, *CFT* clot formation time, *A10* clot firmness amplitude 10 min after CT, *MCF* maximal clot firmness; variables are shown as median (interquartile range), *RI* institutional reference intervals; bold font, median value outside of the RI; *, significant different to T_0_ (*P* < 0.05)

### Osmolarity, hematocrit and platelet counts

No significant difference was found in osmolarity, hematocrit or platelet counts between dogs that received mannitol and those that received HTS at any time point (Table 1). Within group comparisons revealed a significant increase in osmolarity at T_5_, T_60_ and T_120_ after HTS and at T_5_ and T_60_ after mannitol (Table 1).

### Platelet function analysis

At T_0_, Ct_PFA_ was significantly longer in the HTS group than in the mannitol group (*P* = 0.03) but median values were within the RI (Table 2). After treatment, median values were increased above the RI at T_5_ (both groups) and T_60_ (HTS group). However, no significant difference in percentage relative to values at T_0_ was found between the groups at T_5_, T_60_, or T_120_ (Table 3) and no difference was found between measurement times within either group (Table 2).

**Table 3:** Rotational thromboelastometry and platelet function analysis closure time (Ct_PFA_) prior to (T_0_), and at 5 (T_5_), 60 (T_60_) and 120 (T_120_) minutes after administration of mannitol and hypertonic saline (HTS) shown as the percentage of values relative to T_0_

		Osmotherapeutic solution		
Variable	Time point	Mannitol (% from baseline)	HTS (% from baseline)	*P* value
Ct_PFA_	T_5_	130 (103-171)	116 (89-151)	0.31
	T_60_	109 (95-126)	112 (89-149)	1.00
	T_120_	100 (93-109)	91 (87-107)	0.32
EXTEM^®^ CT	T_5_	92 (51-113)	91 (60-239)	0.35
	T_60_	107 (70-128)	95 (60-1299	0.21
	T_120_	113 (40-159)	98 (48-143)	0.65
EXTEM^®^ CFT	T_5_	115 (73-246)	118 (84-216)	0.98
	T_60_	111 (62-194)	113 (83-359)	0.97
	T_120_	115 (80-182)	109 (70-162)	0.62
EXTEM^®^ A10	T_5_	97 (61-122)	98 (16-112)	0.44
	T_60_	103 (86-2014)	102 (88-260)	0.68
	T_120_	97 (92-127)	100 (84-215)	0.37
EXTEM^®^ α-angle	T_5_	98 (65-273)	96 (20-104)	0.48
	T_60_	99 (71-119)	98 (71-109)	0.97
	T_120_	100 (71-113)	100 (93-111)	0.87
EXTEM^®^ MCF	T_5_	97 (70-300)	97 (28-104)	0.86
	T_60_	100 (91-469)	98 (46-106)	0.12
	T_120_	101 (90-446)	97 (89-111)	0.22
FIBTEM^®^ CT	T_5_	100 (70-138)	86 (56-108)	**0.01**
	T_60_	111 (72-163)	81 (55-110)	**0.01**
	T_120_	94 (75-142)	85 (25-126)	0.11
FIBTEM^®^ A10	T_5_	85 (62-167)	103 (15-167)	0.05
	T_60_	114 (82-146)	100 (33-300)	0.76
	T_120_	106 (81-135)	100 (64-200)	0.76
FIBTEM^®^ α-angle	T_5_	101 (80-109)	106 (97-119)	0.09
	T_60_	99 (92-107)	101 (22 (124)	0.60
	T_120_	100 (93-104)	96 (83-112)	0.56
FIBTEM^®^ MCF	T_5_	89 (58-188)	100 (19-171)	0.11
	T_60_	88 (59-104)	100 (14-143)	0.07
	T_120_	88 (62-188)	104 (19-143)	0.60

*Ct*_*PFA*_ platelet closure time, *CT* clotting time, *CFT* clot formation time, *A10* amplitude after 10 min, *MCF* maximal clot firmness; values shown as median (interquartile range) percentage of measurements relative to T_0; bold font, *P* <0.05_

### ROTEM® analysis

No significant difference was found for any parameter between the groups at T_0_ (Table 2) and no significant difference between the groups in percentage relative to T_0_ was found for any parameter except for a shortening of FIBTEM® CT at T_5_ and T_60_ after HTS (Table 3). No significant difference was found between measurement times within either group except for a decrease of EXTEM® MCF at T_5_ and FIBTEM® MCF at T_5_ and T_60_compared to T_0_ in dogs receiving mannitol, and a decrease of FIBTEM® CT at T_5_, T_60_, and T_120_ compared to T_0_ in dogs receiving HTS (*P* < 0.05). Median values where within RI at all time points in both groups.

## Discussion

The present study evaluated the effects on coagulation of intravenous 20% mannitol and 7.2% HTS in a cohort of dogs with suspected ICH using ROTEM® and PFA® analyses. Only minimal differences in coagulation parameters were found between dogs treated with mannitol and those receiving HTS at currently recommended doses. Indeed, a significant difference between the groups in percent of values relative to baseline (T_0_) was only found for FIBTEM® CT, which showed a shorter time until clot detection at T_5_ and T_60_ in dogs receiving HTS. This was mirrored by a shorter FIBTEM® CT at T_5_, T_60_, and T_120_ compared to T_0_ in the HTS group. Furthermore, a short-lived decrease in EXTEM® and FIBTEM® MCF was observed after mannitol. However, median ROTEM® values remained within institutional RIs at all time points in both groups. In contrast, PFA® values increased above institutional RIs after mannitol (T_5_) and HTS (T_5_ and T_60_), although no significant differences were found between groups or time points. In terms of the importance of the detected changes, alterations in FIBTEM® CT are most likely of no relevance, as in human medicine only the clot firmness variables (A5, A10, etc., and MCF) of the FIBTEM® assay are used (eg., for deciding on the replacement of fibrinogen sources) [25]. The very mild changes in MCF and Ct_PFA_ may be signs for a mannitol induced, short-lived fibrin polymerization defect (decrease in MCF FIBTEM®) as well as short lived platelet-fibrin interaction defect, such as impairment of GPIIb/IIIa receptor mediated binding (decrease in MCF EXTEM® combined with impaired platelet function) which might become more prominent after higher doses. Furthermore, plasma osmolarity was significantly increased by 15–25 mOsm/L for up to 1 h (mannitol group) and up to 2 h (HTS group), respectively, but no difference between the groups was detected.

Previous in vitro studies in humans and dogs indicate that both mannitol and HTS affect primary and secondary hemostasis in a dose-dependent fashion by delayed clot formation and impairment of fibrin clot firmness [12, 14, 16, 17]. Indeed, a 1:22 dilution of whole blood with 7.2% HTS (mimicking a dose of 4 ml/kg) significantly affected Ct_PFA_ and ROTEM® EXTEM® CFT and MCF in dogs [16]. Moreover, 20% mannitol affected Ct_PFA_ and ROTEM® EXTEM® variables to a greater extent than equimolar 3% HTS in dilutions mimicking recommended clinical doses [17]. However, given the in vitro nature of these studies and absence of the endothelium and compensatory mechanisms, such as buffering, electrolyte homeostasis, and metabolic degradation and excretion of the drug, results may not reflect in vivo conditions [26]. Moreover, in vitro dilution of blood may result in a more significant dilution of coagulation factors than the corresponding in vivo dose, impacting the kinetics of clot formation. Finally, effects of transendothelial fluid shifts, which are a crucial effect of osmotherapy, are essentially eliminated by in vitro studies.

The findings of the present study only partially confirm recent in vitro findings [17]. The less pronounced effect of mannitol in the present study may to some extent be due to disparate osmolarities of the HTS solutions evaluated (3% HTS in the previous in vitro study compared to 7.2% in the present study). Indeed, hyperosmotic stress may result in impaired enzymatic function in the clotting cascade, slower clot formation and a weaker clot [9, 10, 12]. In the present study no significant difference in plasma osmolarity was found between the two groups despite the higher osmolarity and more rapid administration of HTS. Likewise, no significant difference in plasma osmolarity was found between 7.2% NaCl/HES 200/0.5 and 15% mannitol in a previous study in 40 adult neurosurgical patients [27]. This may, in part, explain the lack of difference in ROTEM® and PFA® parameters between the two osmotherapeutic groups. However, the extent to which the changes observed in ROTEM® and PFA® parameters were due to increased osmolarity, increased sodium load, or to additional hemostatic disturbances from each hyperosmolar molecule itself remains unclear. Lastly, the volume administered in the present study was lower than dilutions used in some previous in vitro studies, which may have masked potential coagulation impairing effects at higher doses.

Similar to the current findings, in two recent studies in people undergoing elective craniotomy or suffering from traumatic brain injury, respectively, no difference in standard coagulation tests and ROTEM® analysis was found between patients administered 20% mannitol and those receiving 3% HTS [15, 28].

To the authors’ knowledge, the present study is the first investigation evaluating platelet function with PFA® after intravenous administration of mannitol or HTS in dogs. Despite the 30% increase in Ct_PFA_ after mannitol at T_5_ and some PFA values above the RI in both groups, the changes did not reach statistical significance and returned to normal within the two-hour study period. A clear advantage of one of the osmotherapeutics to avert platelet dysfunction was therefore not evident, although mannitol may have more pronounced but shorter lived effects compared to HTS.

In contrast to the aforementioned in vitro studies [16, 17], dogs in the present study were not healthy as they all were affected by conditions causing suspected ICH. Of the included dogs, 53% had intracranial neoplasia, albeit equally distributed between both treatment groups. Furthermore, 5 dogs (*n* = 3, HTS group; *n* = 2, mannitol group) included in the study had received one dose of glucocorticoids (≤1 mg/kg) within 7 days prior to the study, which may be expected to increase clot strength and decrease clot lysis in thromboelastography [29]. However, effects on thromboelastographic amplitude and clot lysis in the respective study were found after two 2 and 4 days of treatment, respectively, with an immunosuppressive dose of prednisone (median dose 2.07 mg/kg per 24 h). In contrast, the dogs in the present study received prednisolone in a lower dose and only once. Nevertheless, given heterogeneous diseases and previous treatments, some dogs included in this study were expected to have some abnormal initial PFA® or ROTEM® measurements. Indeed, MCF values were above the RI in EXTEM® (*n* = 1) and FIBTEM® (*n* = 5). This confounder was cancelled out in the evaluation of differences between time points by using the percentage of parameters relative to those measured at T_0_ instead of absolute values, allowing evaluation of a cohort that truly represents the target population of dogs receiving osmotherapy. Given the small numbers of dogs affected by different diseases, no analyses of associations between disease and coagulation parameters was performed and further studies are needed to establish whether certain conditions leading the ICH are associated with coagulation abnormalities in dogs.

The current guidelines for viscoelastic coagulation testing recommend blood sampling for ROTEM® by atraumatic puncture of the jugular vein [30]. However, as compression of the jugular vein is not recommended in dogs with ICH, samples were taken from the lateral saphenous veins in the present study [18]. The extent to which this may have affected results is not clear.

Artefactual hypercoagulability using ROTEM® analysis has been shown to occur due to low red blood cell mass [31, 32]. Likewise, low hematocrits and low platelet count was found to prolong PFA® results [23]. In the present study, some dogs were slightly anemic following administration of osmotherapeutics but hematocrits did not fall below 0.30 L/L and platelet counts not fall below 100 × 10^9^/L in any dog at any time point, which is within the current recommendations for accurate PFA® testing [23].

The current study has potential limitations. Firstly, simultaneous standard coagulation testing (prothrombin time (PT), partial thromboplastin time (aPTT), and fibrinogen concentration) was not assessed. Although ROTEM® analysis enables dynamic assessment of whole blood coagulation and platelet function, standard coagulation testing may have helped in the interpretation of the clinical relevance of findings. However, both PT and aPTT are essentially limited to answering the question whether any intervention exerts effects on thrombin generation, depending on the coagulation factors reflected in the respective assay. As we were not expecting the interventions in our study to exert impacts on thrombin generation, the information obtainable by additional PT and aPTT assays would be limited to detect potential effects induced by dilution. Furthermore, both poor and good associations between standard coagulation tests and ROTEM® analyses have been reported [33, 34], and information about platelet function, fibrin polymerization, and platelet interaction with fibrin is not provided by plasmatic coagulation tests. Further, although there are no results of any conventional fibrinogen assay available, variables of clot firmness (MCF and A10) of ROTEM® FIBTEM® assays are highly correlated with the fibrinogen concentration [31] and adequately provide information about potential fibrin-polymerization defects that are not detectable using standard lab tests. Even if potentially elevated fibrinogen concentrations in some of the dogs might have alleviated coagulation impairment, importantly no relevant alteration of fibrin polymerization was found after osmotherapy in the present study.

Another limitation was that some dogs received additional crystalloid fluid therapy during the study period as treatment was largely at the discretion of the clinicians. Previous in vitro studies in dogs demonstrated a dilutional coagulopathy caused by 0.9% saline on viscoelastic coagulation and PFA® testing [16, 35]. Contrariwise, a recent in vivo study in healthy anesthetized dogs administered with a 15 ml/kg-bolus of isotonic buffered crystalloids did not led to relevant ROTEM® and PFA® abnormalities [36]. Nevertheless, simultaneous crystalloid fluid therapy might have impacted results in individual dogs in the present study. Lastly, although the previous in vitro study evaluated a 3% HTS solution, dogs in the present study were given a 7.2% HTS solution as this was the established institutional treatment protocol for dogs with ICH. It is likely that more significant differences between groups would have been found had mannitol been compared with 3% HTS instead of 7.2% HTS.

## Conclusion

In conclusion, results of this pilot study suggest that mannitol and HTS do not differ in their effects on coagulation when administered in the currently recommended doses in dogs with suspected ICH. Moreover, no clinically relevant impairment of whole blood coagulation was found following treatment with either solution, whereas a short-lived impairment of platelet function was found after both solutions. Further studies are warranted before recommendations can be made with regards to treatment of individual animals.

## Abbreviations

A10: Amplitude after 10 min
CFT: Clot formation time
CT: Clotting time
Ct_PFA_: Platelet function analysis closure time
HTS: Hypertonic saline
ICH: Intracranial hypertension
MCF: Maximum clot firmness
PFA®: Platelet function analyzer
RI: Reference intervals
ROTEM®: Rotational thromboelastometry
